# Immigrant Older Adults: Is Diversity a Challenge for a Global Initiative?

**DOI:** 10.1093/geroni/igaa057.2984

**Published:** 2020-12-16

**Authors:** Noriko Toyokawa, Vivian Lou

## Abstract

Although the global community has discussed needs for establishing international standards of health care for immigrant older adults for decades, it is challenging for policy makers to consider international standards that could meet diverse needs for older adults from various migrant groups. The purpose of this symposium is to discuss challenges and possible strategies to develop global standards to protect immigrant older adults. There will be four presentations on the topic of various needs of older adults from different migrant groups. Noriko Toyokawa will present a study in diversity in parents’ expectations on filial piety among immigrant older adults from different racial/ethnic groups in the Southern California. Weiyu Mao and her colleagues will present their study in the perceived neighborhood cohesion as a protective factor for older Chinese immigrants’ oral health. Allen Glicksman and his colleagues will report the diversity across migrant groups and State Policies that create a challenge in using finding to establish global standards for best practices with older migrants based on a series studies on Mandarin speaking Chinese and Puerto Rican older immigrants. Finally, Mika Marumoto will suggest the ‘reframing of aging initiative’ as a possible means of leading the way of cultivating transformative solutions. Vivian Lou will comment on each presentation, discuss common themes among the presented studies, and address future research directions. With the audience, the presenters will discuss challenges in dealing with diversity issues and suggestions for a global initiative to protect human rights and health care accessibilities for immigrant older adults.

